# Use of the VerifyNow point of care assay to assess the pharmacodynamic effects of loading and maintenance dose regimens of prasugrel and ticagrelor

**DOI:** 10.1007/s11239-021-02386-7

**Published:** 2021-02-13

**Authors:** Dominick J. Angiolillo, Latonya Been, Marc Rubinstein, Michael Martin, Fabiana Rollini, Francesco Franchi

**Affiliations:** 1 University of Florida College of Medicine-Jacksonville, 655 West 8th Street, Jacksonville, FL 32209 USA; 2 Instrumentation Laboratory, San Diego, CA USA

**Keywords:** Platelet function, Prasugrel, Ticagrelor

## Abstract

Prasugrel and ticagrelor are potent oral platelet P2Y_12_ inhibitors and are recommended over clopidogrel in patients with acute coronary syndrome (ACS). Oral platelet P2Y_12_ inhibitors are characterized by varying degrees of pharmacodynamic response profiles as assessed by a variety of commercially available assays. Because of its ease of use, rapid turnaround times and ability to provide results specific to P2Y_12_ inhibitory effects, VerifyNow has emerged as one of the most commonly utilized platelet function assays. However, reference ranges with VerifyNow have been reported mainly for clopidogrel and there has not yet been any study specifically conducted to provide the expected on treatment reference ranges following administration of prasugrel and ticagrelor. This was a prospective single center investigation conducted in 120 patients with ACS who were treated with prasugrel or ticagrelor as per standard of care. Patients who underwent percutaneous coronary interventions (PCI) were treated with a loading dose of prasugrel (60 mg) or ticagrelor (180 mg), and patients who were on maintenance therapy were taking prasugrel (10 mg qd or 5 mg qd) or ticagrelor (90 mg bid). Platelet function testing was performed using the VerifyNow™ PRUTest™. The overall range of PRUTest values was lower than that observed in studies of patients treated with clopidogrel. The use of a maintenance dose regimen had a wider range of PRUTest values compared to the use of a loading dose for both prasugrel (1–179 vs. 2–128) and ticagrelor (1–196 vs. 1–177). The average PRUTest values in patients on prasugrel and ticagrelor maintenance dosing were 20% and 9% higher those observed in patients treated with a loading dose. PRUTest results following loading dose administration were very similar between drugs, but were 20% higher with prasugrel compared with ticagrelor during maintenance dosing. This study establishes expected PRUTest ranges for patients taking loading and maintenance doses of prasugrel and ticagrelor.

*Clinical Trial Registration* http://www.clinicaltrials.gov Unique Identifier: NCT04492423, registered July 2020 retrospectively registered.

## Introduction

Dual antiplatelet therapy (DAPT) with aspirin and a P2Y_12_ inhibitor is the standard of care for the prevention of recurrent atherothrombotic events in patients with an acute coronary syndrome (ACS) [1]. Clopidogrel, prasugrel and ticagrelor are P2Y_12_ inhibitors approved for clinical use in ACS patients [2]. However, clopidogrel-induced antiplatelet effects are subject to broad variability in individual response with a considerable number of patients persisting with high on-treatment platelet reactivity (HPR) [3]. Importantly, HPR is an established marker of thrombotic risk particularly among patients undergoing percutaneous coronary interventions (PCI) [4]. Ticagrelor and prasugrel are newer generation potent P2Y_12_ inhibitors, both are characterized by more robust and less variable antiplatelet effects compared with clopidogrel [2]. Moreover, outcome studies conducted in patients with ACS have shown a greater reduction in recurrent atherothrombotic events with prasugrel and ticagrelor compared with clopidogrel, albeit at the expense of increased bleeding [5]. Accordingly, guidelines recommend the use of prasugrel and ticagrelor over clopidogrel in patients with ACS, particularly in the absence of high bleeding risk [6]. The clinical implications associated with different levels of platelet reactivity induced by platelet P2Y_12_ inhibitors has fueled the interest of developing assays able to detect their effect [4, 7]. Although several assays are commercially available, many of these may be labor intensive and require technical laboratory expertise [4, 7]. Hence, an assay which is easy to use and provides prompt and reliable results is key towards their acceptance and routine implementation in clinical practice [4].

VerifyNow uses a whole blood sample to measure platelet reactivity by assessing the rate and extent of changes in light transmittance caused by platelets aggregating in the presence of agonists that are specific to define the effects of a given antiplatelet agent [7, 8]. In light of its ease of use, rapid turnaround times and ability to provide results specific to P2Y_12_ inhibitory effects, VerifyNow has emerged as one of the most commonly utilized platelet function assays in clinical trials as well as in real-world practice [4, 7]. However, despite its broad utilization in patients treated with all commercially available P2Y_12_ inhibitors, reference ranges with VerifyNow have been reported mainly for clopidogrel and there has not yet been any study specifically conducted to provide the expected on treatment reference ranges following administration of prasugrel and ticagrelor [9]. The ever-growing use of these agents underscores the importance of defining these ranges to help guide practitioners in the management of their patients treated with prasugrel or ticagrelor if testing with VerifyNow is performed. The primary aim of this study was to determine the range of expected values of platelet reactivity measured by VerifyNow in patients with ACS following acute and chronic administration of prasugrel or ticagrelor.

## Methods

### Study design and participants

This was a prospective single center investigation conducted in male and female adult (> 18 years of age) patients with an ACS (*n* = 120) treated with prasugrel or ticagrelor as per standard of care (Clinicaltrials.gov identifier: NCT04492423). The study was performed at the University of Florida Health-Jacksonville (Jacksonville, FL, USA), and approved by Western Institutional Review Board (WIRB). Patients with ACS included those with a non-ST elevation ACS (NSTE-ACS), including unstable angina and non-ST elevation myocardial infarction, and ST elevation myocardial infarction (STEMI). Written informed consent was obtained from all individual participants included in the study prior to their enrollment. Assessments included patients undergoing PCI and treated with a loading dose of prasugrel (60 mg) or ticagrelor (180 mg) as well as patients who were on maintenance therapy with prasugrel (10 mg qd or 5 mg qd) or ticagrelor (90 mg bid). Loading and maintenance doses were administered as uncrushed integral tablets. All patients were on a background of aspirin therapy (325 mg loading dose in patients undergoing PCI followed by 81 mg qd). Key exclusion criteria were inability to provide written informed consent, subjects treated with an investigational antiplatelet agent, use of a glycoprotein IIb/IIIa inhibitor within past 2 weeks, treatment with any therapy containing dipyridamole within past 2 weeks, and women who were pregnant or of child bearing potential. The study complied with the Declaration of Helsinki, was approved by the Western Institutional Review Board and all patients gave their written informed consent.

### Blood sampling

Peripheral venous blood samples were drawn through a short venous catheter inserted into an arm vein and collected in citrated tubes as appropriate for assessments. The first 2–4 mL of blood were discarded to avoid spontaneous platelet activation. For all enrolled patients undergoing PCI, duplicate samples were collected and assayed between 2 and 6 h post loading or maintenance dose administration. Blood samples were drawn in conjunction with routine testing performed as part of standard patient care. All blood samples were assayed in duplicate between 10 min and 1 h following sample collection. A complete blood count measurement was performed for each enrolled subject from a sample collected at the time of blood draw or within 1 week.

### Platelet function testing

**Fig. 1:**
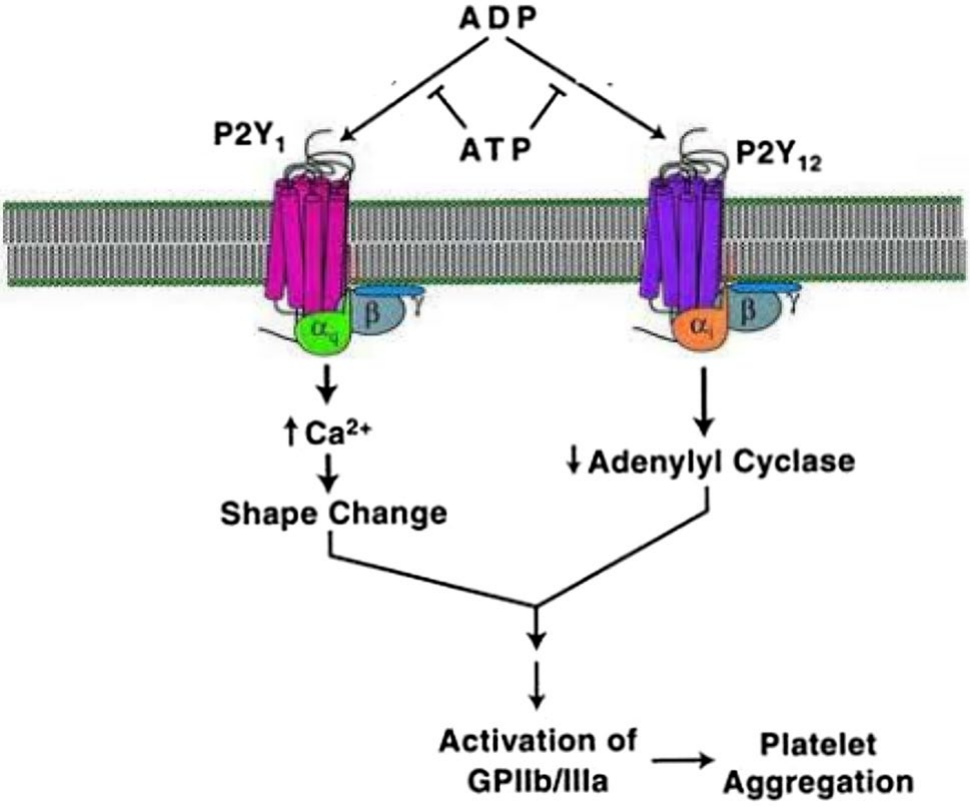
Platelet activation mediated by platelet P2Y_1_ and P2Y_12_ receptors. Illustration of the differences between P2Y_1_ and P2Y_12_ mediated signaling and the selective nature of the VerifyNow test in defining the effects of P2Y_12_ inhibitors Adapted with permission from Nicholas RA (2001) Identification of the P2Y12 receptor: a novel member of the P2Y family of receptors activated by extracellular nucleotides. Mol Pharmacol, 60(3):416–420

Platelet Function Testing was performed using VerifyNow PRUTest (Accriva Diagnostics, wholly owned by Instrumentation Laboratory, San Diego, USA). Platelet activation induced by adenosine diphosphate (ADP) is mediated by two receptors located on platelets, P2Y_1_ and P2Y_12_ [10]. As depicted in Fig. 1, both receptors are activated by ADP and lead to the final common pathway that mediates platelet aggregation, i.e., activation of glycoprotein IIb/IIIa receptors. VerifyNow is a turbidimetric based optical detection system, which measures platelet-induced aggregation. The VerifyNow PRUTest is designed to measure platelet aggregation mediated by P2Y_12_ receptor blockade. The VerifyNow PRUTest is based upon the ability of activated platelets to bind fibrinogen. Fibrinogen-coated microparticles aggregate in whole blood in proportion to the number of expressed platelet GP IIb/IIIa receptors. Results are reported as P2Y_12_ Reaction Units (PRU) based on the rate and extent of aggregation, which reflects the amount of P2Y_12_ receptor-mediated aggregation specific to platelets. A PRU result is calculated based upon the rate and extent of platelet aggregation recorded in the channel containing the platelet agonist, ADP. P2Y_12_ receptor mediated platelet aggregation and PRUTest results from VerifyNow are not influenced by non-specific platelet aggregation mediated through P2Y_1_. To accomplish the goal of having a test with reduced non-specific aggregation, the VerifyNow PRUTest uses prostaglandin E_1_ (PGE_1_) in addition to ADP to make the test more sensitive and specific for the effects of ADP mediated by the P2Y_12_ receptor. A PRU > 208 identifies patients with HPR, an established marker of increased thrombotic risk [4].

### Statistical analysis

Results were analyzed per Clinical and Laboratory Standards Institute (CLSI) Guideline (“Defining, Establishing and Verifying Reference Intervals in the Clinical Laboratory”) [11]. The per treatment evaluable population consisted of subjects who were enrolled and had no pre-specified protocol deviations. Data from the patients not meeting study entry criteria after blood sample collection were excluded from the analysis. Continuous variables are expressed as mean, standard deviation, 25th and central 95th percentile reference limits (RL), 90% confidence intervals (CI), and range. Categorical variables are expressed as number (*N*), standard deviation (SD), correlation coefficient (Pearson’s *r*) and percentage (%) of total for the ticagrelor and prasugrel populations. There was no imputation for missing data. The study was not designed for statistical comparisons between the different cohorts and accordingly descriptive reporting of data are provided.

## Results

### Baseline characteristics

A total of 130 male and female adult patients receiving aspirin plus prasugrel or ticagrelor were enrolled. A total of 10 patients were excluded from analysis due to protocol deviations which included lack of baseline complete blood count (*n* = 5), study procedure violation (*n* = 4) and incorrect P2Y_12_ inhibitor (*n* = 1). Therefore, a total of 120 patients were eligible for analysis. The mean age for all participating patients was 59.8 (9.7), with a range of 34 to 70 years. Prasugrel was used in 63 (53%) of patients; 28 (44%) of these received a loading dose of 60 mg in the catheterization laboratory; the remaining 35 (56%) were on either a 5 mg (*n* = 4) or 10 mg (*n* = 31) qd maintenance dose at the time of enrollment. Ticagrelor was used in 57 (47%) of patients; 32 (56%) of these received a loading dose of 180 mg in the catheterization laboratory; the remaining 25 (44%) were on a 90 mg bid maintenance dose at the time of enrollment. A detailed description of baseline demographics is provided in Table 1.

**Table 1:** Baseline and demographic data

Parameter	Prasugrel	Ticagrelor	All
Evaluable subjects	63	57	120
Male—N (%)	41	37	78
Female—N (%)	22	20	42
Mean age (SD)—N (%)	59.7 (9.5)	59.9 (10.1)	59.8 (9.7)
Age ≥ 65	17	22	39
Ethnicity—N (%)	63 (100)	57 (100)	120 (100)
White	49 (77.8)	35 (61.4)	84 (70.0)
Asian	0 (0)	0 (0)	0 (0)
Hispanic or Latino	2 (3.2)	3 (5.3)	5 (4.2)
Black or African American	12 (19.0)	19 (33.3)	31 (25.8)
Other	0 (0)	0 (0)	0 (0)
Smoking—N (%)	12 (19.0)	8 (14.0)	20 (16.7)
Diabetes mellitus—N (%)	22 (34.9)	18 (31.6)	40 (33.3)
Hyperlipidemia—N (%)	35 (55.6)	33 (57.9)	68 (56.7)
Hypertension—N (%)	51 (81.0)	40 (70.2)	91 (75.8)
Prior MI p—N (%)	33 (52.4)	33 (57.9)	66 (55.0)
Prior CABG—N (%)	10 (15.9)	8 (14.0)	18 (15.0)
Prior PCI—N (%)	39 (61.9)	39 (68.4)	78 (65.0)
PAD—N (%)	2 (3.2)	4 (7.0)	6 (5.0)
Prior CVA—N (%)	0 (0)	8 (14.0)	8 (6.7)
Mean BMI kg/M^2^ (SD)	32.3 (8.6)	30.0 (5.9)	31.2 (7.4)
Mean hematocrit, % (SD)	40.3 (4.2)	38.2 (5.7)	39.3 (5.1)
Mean platelets × 10^3^ (SD)	227 (56.4)	237 (67.7)	232 (62)
Mean hemoglobin, g/dL (SD)	13.4 (1.4)	12.7 (2.2)	13.1 (1.9)

### VerifyNow testing

The average time from prasugrel loading dose to PRUTest blood draw was 4 h 46 min, range 3 h 48 min to 8 h 38 min, median 4 h 21 min. The average time from last prasugrel maintenance dose to PRUTest blood draw for these patients was 3 h 9 min, range 2 h 1 min to 5 h 47 min, median 3 h 6 min. The average time from ticagrelor loading dose to PRUTest blood draw was 4 h 48 min, range 1 to 7 h and 10 min, median 4 h 8 min. The average time from last ticagrelor maintenance dose to PRUTest blood draw for these patients was 3 h 53 min, range 2 h 10 min to 5 h 20 min, median 3 h 40 min.

**Fig. 2:**
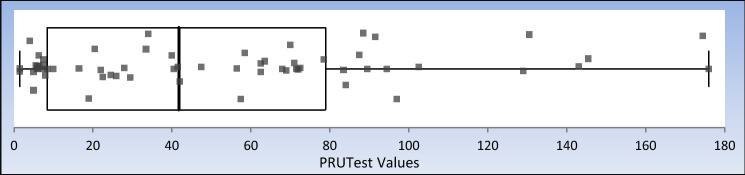
Distribution of on treatment PRUTest values among prasugrel treated patients. Box Whisker plot of the minimum, median, maximum, interquartile range and outliers of PRUTest values in prasugrel treated patients

**Fig. 3:**
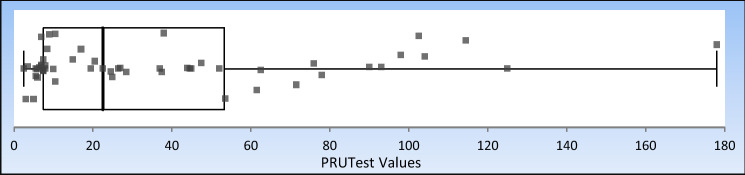
Distribution of on treatment PRUTest values among ticagrelor treated patients. Box Whisker plot of the minimum, median, maximum, interquartile range and outliers of PRUTest values in ticagrelor treated patients

The range of PRUTest values for patients on prasugrel was 1–196 with an average of 52.3**,** with Pearson’s correlation coefficient of duplicate measurements of 0.96–0.97 (Table 2). Box–Whisker distribution plots of individual patient data of prasugrel treated patients are illustrated in Fig. 2. Ranges of PRUTest values were 1–177 with an average of 45.6 for patients treated with a loading dose and 1–196 with an average of 57.8 for those on maintenance dosing. The range of PRUTest values for patients on ticagrelor was 1–179 with an average of 42.2 (Table 3), with Pearson’s correlation coefficient of duplicate measurements of 0.94–0.99. Box–Whisker distribution plots of individual patient data of ticagrelor treated patients are illustrated in Fig. 3. Specifically, ranges of PRUTest values were 2–128 with an average of 43.7 for patients treated with a loading dose and 1–179 with an average of 40.3 for those on maintenance dosing (Table 3).

**Table 2:** Prasugrel PRUTest data

All		5 to 10 mg		60 mg	
N	63	N	35	N	28
Average	52.3	Average	57.8	Average	45.6
SD	44.70	SD	44.3	SD	45.0
5th Percentile	5	5th Percentile	5.7	5th Percentile	4.4
25th Percentile	8.88	25th Percentile	20.9	25th Percentile	7.1
95th Percentile	142.38	95th Percentile	135.8	95th Percentile	126.9
Range	1–196	Range	1–196	Range	1–177
Pearson’s r	0.96	Pearson’s r	0.97	Pearson’s r	0.96

**Table 3:** Ticagrelor PRUTest data

All		90 mg		180 mg	
N	57	N	25	N	32
Average	42.2	Average	40.3	Average	43.7
SD	54.2	SD	42.4	SD	62.8
5th Percentile	4.625	5th Percentile	4.2	5th Percentile	5.3
25th Percentile	7.5	25th Percentile	10.5	25th Percentile	7.0
95th Percentile	117.1	95th Percentile	111.2	95th Percentile	114.5
Range	1–179	Range	1–179	Range	2–128
Pearson’s r	0.98	Pearson’s r	0.99	Pearson’s r	0.94

A total of 2 patients (prasugrel, *n* = 1; ticagrelor, *n* = 1) had HPR status. The prasugrel treated patient was confirmed to be compliant with a 10 mg maintenance dose regimen and had a PRUTest value of 257 with the last dose taken was 2 h and 19 min prior to the study blood draw. The ticagrelor treated patient received a 180 mg loading dose and had a PRUTest value of 322 at 5 h and 26 min following drug administration. These patients were included in the per treatment evaluable group for statistical evaluation.

## Discussion

The use of platelet P2Y_12_ inhibiting therapy, in adjunct to aspirin, is the cornerstone of treatment for patients with an ACS [1]. Clopidogrel, prasugrel and ticagrelor are P2Y_12_ inhibitors approved for clinical use in ACS patients [2]. However, in the absence of contraindications, guidelines recommend the use of prasugrel and ticagrelor over clopidogrel in light of their superior clinical outcomes [1]. In particular, both prasugrel and ticagrelor are associated with a greater reduction in recurrent ischemic events, albeit at the expense of increased bleeding, but with a net benefit profile which still favors their use over clopidogrel [5, 6]. These findings are attributed to the more potent platelet inhibitory effects of prasugrel and ticagrelor over clopidogrel [2]. Clopidogrel in fact is well known to be associated with broad variability in platelet inhibitory effects with a considerable number of patients persisting with HPR, an established marker of thrombotic risk [3, 4]. Studies associating HPR with adverse outcomes have been mostly conducted among clopidogrel treated patients [4]. Although a number of assays have been used to support this association, most data are derived from the use of the VerifyNow [4]. This is due to its ease of use, rapid turnaround times and ability to provide results specific to P2Y_12_ inhibitory effects [4, 7]. However, reference ranges with the VerifyNow have been reported mainly for clopidogrel and, despite its broad utilization in patients treated with all commercially available P2Y_12_ inhibitors, there has not yet been any study specifically conducted to provide the expected on treatment reference ranges following administration of prasugrel and ticagrelor. For these reasons, it is important to determine the degree at which these newer generation P2Y_12_ inhibitors impact platelet function and therefore PRUTest values. This cannot be established without the benefit of a carefully conducted study, using proven methods in the intended population for P2Y_12_ inhibitor therapy.

Our findings demonstrate the following: (a) the overall range of PRUTest values is lower than that observed in studies of patients treated with clopidogrel [9, 12–14]; (b) in both patients treated with prasugrel and those treated with ticagrelor, the use of a maintenance dose regimen had a wider range of PRUTest values compared to the use of a loading dose (1–179 vs. 2–128 for prasugrel and 1–196 vs. 1–177 for ticagrelor); (c) the average PRUTest values in patients on prasugrel and ticagrelor maintenance dosing were 20% and 9% higher those observed in patients treated with a loading dose; (d) PRUTest results following loading dose administration were very similar between drugs, but were 20% higher with prasugrel compared with ticagrelor during maintenance dosing.

Overall, the results of our investigation provide important insights on anticipated ranges of PRUTest values among patients treated with the newer generation P2Y_12_ inhibitors prasugrel and ticagrelor both following loading dose and maintenance dose administration [15, 16]. The PRUTest values are in line with the greater potency of these agents compared with clopidogrel [15, 16]. Indeed, the presence of a variability in platelet inhibitory effects is consistent with orally administered drugs which were not developed to cause complete suppression of P2Y_12_ mediated signaling like that achieved with intravenous therapy (i.e., cangrelor) [17–20]. Nevertheless, they do support the concept that patients treated with these agents have enhanced platelet inhibition which fall well below previously defined thresholds of HPR associated with increased thrombotic risk [4]. In fact, only 1 patient per group was identified to have HPR status. The mechanisms associated with this HPR are unknown, but most likely to be associated with impaired drug absorption or metabolism. Pharmacokinetic assessments would have been useful to interpret these individual patient findings.

The observation that the differences in levels of platelet reactivity observed following loading and maintenance dose administration were more enhanced with prasugrel than with ticagrelor can be attributed to the regimens being used. In fact, the loading dose of prasugrel is sixfold the maintenance dose (60 mg versus 10 mg). These differences were less marked in dose-finding studies using higher maintenance dose (i.e., 15 mg qd) regimens [21]. Moreover, because prasugrel is administered once daily, PRUTest values can be affected by those patients with higher platelet turnover rates such as patients with diabetes mellitus [22, 23]. In contrast, the loading dose of ticagrelor and the total daily maintenance dose are the same (180 mg). Moreover, ticagrelor maintenance dose is administered twice daily and may thus allow for reduced more consistent platelet inhibitory effects. Although our study was not designed to compare the pharmacodynamic effects of prasugrel versus ticagrelor, the above made observations on the dosing regimens used can contribute to similar PRUTest values between agents following loading dose administration and the lower levels with ticagrelor compared with prasugrel during maintenance dosing. These findings are also consistent with other studies [24, 25].

### Study limitations

The present investigation was conducted to provide the expected on treatment reference ranges following administration of prasugrel and ticagrelor and not to make comparative assessments on the pharmacodynamic effects of these two agents which would have required a larger sample size. Similarly, the limited sample size of this study did not allow for subgroup analysis. Although opioids, commonly used in STEMI, have shown to affect absorption of oral P2Y_12_ inhibitors, their impact on our study results is unknown as information on ACS type and opioid use was not collected [26, 27]. Similarly, hematologic parameters other than hematocrit, platelet count, and hemoglobin were not collected. Moreover, the current study had as objective evaluating on treatment reference ranges following administration of prasugrel and ticagrelor in patients with ACS for whom these agents are recommended [1]. Therefore, our findings cannot be extrapolated to a stable clinical setting where these agents, albeit not recommended for use, are not infrequently used. Ultimately, our study was limited to the investigation of prasugrel and ticagrelor in patients with an ACS and the on treatment reference ranges following administration of other P2Y_12_ inhibitors (i.e., clopidogrel, cangrelor, selatogrel) in this clinical setting were not tested.

## Conclusions

The PRUTest values of ticagrelor and prasugrel in this study reflect the greater potency of these agents compared with clopidogrel. The variability in platelet inhibitory effects, as reflected in PRUTest results, is consistent with orally administered drugs which were not developed for complete suppression of P2Y_12_ medicated signaling. The use the VerifyNow PRUTest assay is well standardized, and makes it a reproducible point of care assay that allows for measuring platelet reactivity and assessing interindividual variability in response to oral P2Y_12_ receptor inhibitors, including prasugrel and ticagrelor, convenient in the acute/in-patient care or outpatient/office settings.

## References

[1] Capodanno D, Alfonso F, Levine GN, Valgimigli M, Angiolillo DJ (2018). ACC/AHA Versus ESC Guidelines on Dual Antiplatelet Therapy: JACC Guideline Comparison. J Am Coll Cardiol.

[2] Franchi F, Angiolillo DJ (2015). Novel antiplatelet agents in acute coronary syndrome. Nat Rev Cardiol.

[3] Angiolillo DJ, Fernandez-Ortiz A, Bernardo E, Alfonso F, Macaya C, Bass TA, Costa MA (2007). Variability in individual responsiveness to clopidogrel: clinical implications, management, and future perspectives. J Am Coll Cardiol.

[4] Sibbing D, Aradi D, Alexopoulos D, Ten Berg J, Bhatt DL, Bonello L, Collet JP, Cuisset T, Franchi F, Gross L, Gurbel P, Jeong YH, Mehran R, Moliterno DJ, Neumann FJ, Pereira NL, Price MJ, Sabatine MS, So DYF, Stone GW, Storey RF, Tantry U, Trenk D, Valgimigli M, Waksman R, Angiolillo DJ (2019). Updated expert consensus statement on platelet function and genetic testing for guiding P2Y12 receptor inhibitor treatment in percutaneous coronary intervention. JACC Cardiovasc Interv.

[5] Wiviott SD, Braunwald E, McCabe CH, Montalescot G, Ruzyllo W, Gottlieb S, Neumann FJ, Ardissino D, De Servi S, Murphy SA, Riesmeyer J, Weerakkody G, Gibson CM, Antman EM (2007). Prasugrel versus clopidogrel in patients with acute coronary syndromes. N Engl J Med.

[6] Wallentin L, Becker RC, Budaj A, Cannon CP, Emanuelsson H, Held C, Horrow J, Husted S, James S, Katus H, Mahaffey KW, Scirica BM, Skene A, Steg PG, Storey RF, Harrington RA (2009). Ticagrelor versus clopidogrel in patients with acute coronary syndromes. N Engl J Med.

[7] Michelson AD (2009). Methods for the measurement of platelet function. Am J Cardiol.

[8] Malinin A, Pokov A, Spergling M, Defranco A, Schwartz K, Schwartz D, Mahmud E, Atar D, Serebruany V (2007). Monitoring platelet inhibition after clopidogrel with the VerifyNow-P2Y12(R) rapid analyzer: the VERIfy Thrombosis risk ASsessment (VERITAS) study. Thromb Res.

[9] VerifyNow PRUTest - Platelet Reactivity Test Device Package Insert www.instrumentationlaboratory.com/us/en/verifynow-system

[10] Storey RF (2006). Biology and pharmacology of the platelet P2Y12 receptor. Curr Pharm Des.

[11] Defining, establishing, and verifying reference intervals in the clinical laboratory; approved guideline, CLSI document EP28-A3, Clinical and Laboratory Standards Institute (CLSI) www.clsi.org

[12] Bae JP, Candrilli SD, Fortenberry J, Meyers JL, Jakubowski JA, Drenning D (2014). Point-of-care platelet reactivity determination with VerifyNow-P2Y12 following administration of clopidogrel or prasugrel: data from a real-world, clinical care inpatient setting. Hosp Pract.

[13] Price MJ, Angiolillo DJ, Teirstein PS, Lillie E, Manoukian SV, Berger PB, Tanguay JF, Cannon CP, Topol EJ (2011). Platelet reactivity and cardiovascular outcomes after percutaneous coronary intervention- a time-dependent analysis of the Gauging Responsiveness with a VerifyNow P2Y12 Assay: impact on thrombosis and safety (GRAVITAS) trial. Circulation.

[14] Stone GW, Witzenbichler B, Weisz G, Rinaldi MJ, Neumann FJ, Metzger DC, Henry TD, Cox DA, Duffy PL, Mazzaferri E, Gurbel PA, Xu K, Parise H, Kirtane AJ, Brodie BR, Mehran R, Stuckey TD (2013). Platelet reactivity and clinical outcomes after coronary artery implantation of drug-eluting stents (ADAPT-DES): a prospective multicentre registry study. Lancet.

[15] Michelson AD, Frelinger AL, Braunwald E, Downey WE, Angiolillo DJ, Xenopoulos NP, Jakubowski JA, Li Y, Murphy SA, Qin J, McCabe CH, Antman EM, Wiviott SD (2009). Pharmacodynamic assessment of platelet inhibition by prasugrel vs. clopidogrel in the TRITON-TIMI 38 trial. Eur Heart J.

[16] Storey RF, Angiolillo DJ, Patil SB, Desai B, Ecob R, Husted S, Emanuelsson H, Cannon CP, Becker RC, Wallentin L (2010). Inhibitory effects of ticagrelor compared with clopidogrel on platelet function in patients with acute coronary syndromes: the PLATO (PLATelet inhibition and patient Outcomes) PLATELET substudy. J Am Coll Cardiol.

[17] Angiolillo DJ, Schneider DJ, Bhatt DL, French WJ, Price MJ, Saucedo JF, Shaburishvili T, Huber K, Prats J, Liu T, Harrington RA, Becker RC (2012). Pharmacodynamic effects of cangrelor and clopidogrel: the platelet function substudy from the cangrelor versus standard therapy to achieve optimal management of platelet inhibition (CHAMPION) trials. J Thromb Thrombol.

[18] Rollini F, Franchi F, Thano E, Faz G, Park Y, Kureti M, Cho JR, Been L, Bass TA, Angiolillo DJ (2017). In vitro pharmacodynamic effects of cangrelor on platelet P2Y(12) receptor-mediated signaling in ticagrelor-treated patients. JACC Cardiovasc Interv.

[19] Rollini F, Franchi F, Tello-Montoliu A, Patel R, Darlington A, Ferreiro JL, Cho JR, Muñiz-Lozano A, Desai B, Zenni MM, Guzman LA, Bass TA, Angiolillo DJ (2014). Pharmacodynamic effects of cangrelor on platelet P2Y12 receptor-mediated signaling in prasugrel-treated patients. JACC Cardiovasc Interv.

[20] Franchi F, Rollini F, Rivas A, Wali M, Briceno M, Agarwal M, Shaikh Z, Nawaz A, Silva G, Been L, Smairat R, Kaufman M, Pineda AM, Suryadevara S, Soffer D, Zenni MM, Bass TA, Angiolillo DJ (2019). Platelet inhibition with cangrelor and crushed ticagrelor in patients with ST-segment-elevation myocardial infarction undergoing primary percutaneous coronary intervention. Circulation.

[21] Jernberg T, Payne CD, Winters KJ, Darstein C, Brandt JT, Jakubowski JA, Naganuma H, Siegbahn A, Wallentin L (2006). Prasugrel achieves greater inhibition of platelet aggregation and a lower rate of non-responders compared with clopidogrel in aspirin-treated patients with stable coronary artery disease. Eur Heart J.

[22] Ferreiro JL, Angiolillo DJ (2011). Diabetes and antiplatelet therapy in acute coronary syndrome. Circulation.

[23] Capodanno D, Angiolillo DJ (2020). Antithrombotic therapy for atherosclerotic cardiovascular disease risk mitigation in patients with coronary artery disease and diabetes mellitus. Circulation.

[24] Rollini F, Franchi F, Cho JR, DeGroat C, Bhatti M, Muniz-Lozano A, Singh K, Ferrante E, Wilson RE, Dunn EC, Zenni MM, Guzman LA, Bass TA, Angiolillo DJ (2016). A head-to-head pharmacodynamic comparison of prasugrel vs. ticagrelor after switching from clopidogrel in patients with coronary artery disease: results of a prospective randomized study. Eur Heart J.

[25] Franchi F, Rollini F, Aggarwal N, Hu J, Kureti M, Durairaj A, Duarte VE, Cho JR, Been L, Zenni MM, Bass TA, Angiolillo DJ (2016). Pharmacodynamic comparison of prasugrel versus ticagrelor in patients with type 2 diabetes mellitus and coronary artery disease: the OPTIMUS (Optimizing Antiplatelet Therapy in Diabetes Mellitus)-4 study. Circulation.

[26] Franchi F, Rollini F, Angiolillo DJ (2017). Antithrombotic therapy for patients with STEMI undergoing primary PCI. Nat Rev Cardiol.

[27] Franchi F, Rollini F, Park Y, Hu J, Kureti M, Rivas Rios J, Faz G, Yaranov D, Been L, Pineda AM, Suryadevara S, Soffer D, Zenni MM, Bass TA, Angiolillo DJ (2019). Effects of methylnaltrexone on ticagrelor-induced antiplatelet effects in coronary artery disease patients treated with morphine. JACC Cardiovasc Interv.

